# Changes in axial length in anisometropic children wearing orthokeratology lenses

**DOI:** 10.3389/fmed.2023.1266354

**Published:** 2023-11-02

**Authors:** Jian Qin, Huiling Qing, Na Ji, Tianbin Lyu, Hui Ma, Menghai Shi, Shiao Yu, Conghui Ma, Aicun Fu

**Affiliations:** ^1^Department of Ophthalmology, Henan Provincial People's Hospital, Henan Eye Hospital, Henan Eye Institute, People's Hospital of Zhengzhou University, Zhengzhou, China; ^2^Department of Ophthalmology, The Affiliated Eye Hospital of Suzhou Vocational Health College, Suzhou, China; ^3^Department of Ophthalmology, The First Affiliated Hospital of Zhengzhou University, Zhengzhou, China

**Keywords:** anisometropia, myopia, unilateral orthokeratology lens, axial length, refractive state

## Abstract

**Purpose:**

There is a particular anisometropia occurring in one eye with myopia, while the other eye has very low myopia, emmetropia, or very low hyperopia. It is unclear how the binocular axial length changes when these children wear unilateral OK lenses only in the more myopic eyes. This study investigates the changes in the axial elongation of both eyes.

**Methods:**

This is a 1-year retrospective study. In total, 148 children with myopic anisometropia were included. The more myopic eyes were wearing orthokeratology lenses (treated eyes), whereas the contralateral eyes were not indicated for visual correction (untreated eyes). The untreated eyes were classified into three subgroups based on the spherical equivalent refraction (SER): low myopia (≤ -0.50 D, *n* = 37), emmetropia (+0.49 to −0.49 D, *n* = 76), and low hyperopia (≥0.50 D, *n* = 35). Changes in the axial length (AL) were compared between the untreated and treated eyes and among the three subgroups.

**Results:**

The axial elongation was 0.14 ± 0.18 mm and 0.39 ± 0.27 mm in all treated and untreated eyes, respectively (*p* < 0.001). The interocular AL difference decreased significantly from 1.09 ± 0.45 mm at the baseline to 0.84 ± 0.52 mm at 1 year (*p* < 0.001). The baseline median (Q1, Q3) SER of the untreated eyes were −0.75 D (−0.56, −0.88 D), 0.00 D (0.00, −0.25 D), and +0.75 D (+1.00, +0.62 D) in low myopia, emmetropia, and low hyperopia subgroups, respectively. The axial elongation was 0.14 ± 0.18 mm, 0.15 ± 0.17 mm, and 0.13 ± 0.21 mm (*p* = 0.92) in the treated eyes and 0.44 ± 0.25 mm, 0.35 ± 0.24 mm, and 0.41 ± 0.33 mm in the untreated eyes (*p* = 0.11) after 1 year. Multivariate linear regression analyses only showed significant differences in axial elongation between the emmetropia and low myopia subgroups of untreated eyes (*p* = 0.04; *p* > 0.05 between other subgroups).

**Conclusion:**

Unilateral orthokeratology lenses effectively reduced axial elongation in the more myopic eyes and reduced interocular AL differences in children with myopic anisometropia. The refractive state of the untreated eyes did not affect the axial elongation of the more myopic eye wearing the orthokeratology lens. In the untreated eyes, AL increased faster in the low myopia subgroup than in the emmetropia subgroup.

## 1. Introduction

Anisometropia is defined as the difference of ≥1.00 D between the spherical equivalents of the two eyes (1). Anisometropia >2.00 D against the rule cylinder and 3.00 D sphere (2) may cause strabismus, amblyopia, binocular vision dysfunction, and asthenopia (3–5). Various measures such as spectacles and contact lenses with different designs were used to correct anisometropia. However, due to the lens effect-related aniseikonia, many anisometropic patients may not tolerate spectacles (6). Traditional rigid gas-permeable contact lenses (7) and single-vision soft contact lenses (8) can decrease aniseikonia and improve binocular vision but cannot prevent the progression of myopia (7, 9). Additionally, some children cannot tolerate the foreign-body sensation caused by daily wearing rigid gas-permeable contact lenses. These are not ideal methods for controlling myopia progression and decreasing anisometropia in children with myopic anisometropia (6–9). Orthokeratology (OK) lenses are specifically designed gas-permeable contact lenses that can not only improve the unaided visual acuity (VA) but also reduce anisometropia and prevent myopia progression in anisometropic patients (10–19). Two 2-year (11, 15) and three 1-year (12–14) retrospective studies found a significant reduction in interocular axial length (AL) difference of unilateral myopic anisometropia (UMA, one eye is myopic and the other eye is emmetropic) children wearing a unilateral OK lens.

Just like UMA children, anisometropic children have more myopia in one eye and very low myopia or very low hyperopia in the other eye. They are common that UMA children wear the OK lenses just in the more myopic eye, while the contralateral untreated eye serve as control. In order to understand the change in AL of both eyes in these children and find reasonable treatments, this study compared the interocular differences in AL and the AL changes in the more myopic eyes wearing the OK lenses during different refractive states of the contralateral untreated eyes. AL changes in the contralateral untreated eyes in three different refractive states were also compared.

## 2. Methods

### 2.1. Participants

Data obtained from the patients' clinical records of 148 children with UMA who visited ^***^ hospital: the First Affiliated Hospital of Zhengzhou University between January 2017 and September 2019 and met the following criteria were retrospectively collected. The inclusion criteria were as follows: patients aged 8–14 years, cycloplegic spherical equivalent refraction (SER) of −1.00 D to −4.50 D in the more myopic eye and +2.00 D to −1.25 D in the contralateral eye, anisometropia of ≥1.0 D, astigmatism of <2.00 D in both the eyes, monocular best corrected VA of 0 (LogMAR charts) or better, intraocular pressure of <21 mmHg, no history of use of medications or contact lenses to control the progression of myopia, no manifest strabismus, no history of other eye diseases and surgery, and the decentration of treatment zone <1 mm during the wearing of OK lenses.

### 2.2. Study design

The more myopic eyes were wearing the OK lenses (treated eyes), whereas and the contralateral eyes were not indicated for visual correction (untreated eyes). The untreated eyes were classified into three subgroups according to their refractive states: low myopia (SER: ≤-0.50 D), emmetropia (SER: +0.49 D to −0.49 D), and low hyperopia (SER: ≥0.50 D) (20, 21). Therefore, the treated eyes were classified into three subgroups according to the refractive state of the untreated eyes (low myopia, emmetropia, or low hyperopia). Four-zone five-curve reverse geometry OK lenses [Boston XO material; oxygen permeability 100 × 10^−11^ (cm^2^/s) (mlO^2^/ml × mmHg); Autek Corporation, Hefei, China] were used. All patients wore the OK lenses continuously for 8–10 h during sleep every day. Routine follow-ups were arranged, and additional unscheduled visits were arranged if necessary.

AL, corneal power, corneal astigmatism, and anterior chamber depth (ACD) were measured using a non-contact partial coherence interferometer (IOLMaster-500, Carl Zeiss, Jena, Germany). Three drops of 1% cyclopentolate (Alcon, Puurs, Belgium) were instilled with 10-min intervals between consecutive administrations. Three autorefraction measurements (ARK-1, Nidek, Japan) were performed 10 min later. The mean values indicated the degree of myopia, which is expressed as SER. OK lenses were replaced at approximately every 1.5 years. However, the OK lens was reordered when the residual SER was > −0.50 D at any visit after treatment stabilization.

Corneal topography with the Topographic Modeling System (TMS-4, Tomey Corporation, Tokyo, Japan) was used to measure the corneal parameters by the same professional technician. A different tangential curvature corneal topography was established by subtracting the pre-OK map from the 1-month post-OK in this study. We adjusted the refractive power change around the corneal vertex to 1.00 D aiming to keep the color of the optical area within two colors. The central blue zone is the treatment zone, and the surrounding red zone is the reverse curve zone. The center of the treatment zone and degree of decentration on the tangential difference map were analyzed with MATLAB. The distance from the pupil center to the center of the treatment zone was considered to be a decentration distance (22).

### 2.3. Statistical analysis

Normal-distributed (mean ± SD) and skew-distributed continuous variables [medians with first (Q1) and third quartiles (Q3)] of baseline parameters were compared using a *t*-test and non-parametric rank-sum test, respectively, and the chi-squared test was used to evaluated categorical variables. The independent sample *t*-test was conducted to assess differences in axial elongation after 1 year between treated and untreated eyes and interocular difference in AL between baseline and 1 year. Analysis of variance (ANOVA) was used to assess the axial elongation after 1 year among different subgroups of the treated and untreated eyes. Furthermore, multivariate linear regression analyses were applied to estimate the axial elongation between untreated and treated eyes, the changes in AL among different subgroups of the treated and untreated eyes, and the reduction in the interocular AL difference. All statistical analyses in this study were performed in software Empower (R; www.empowerstats.com, X & Y Solutions Inc., Boston, MA, USA) and R (http://www.R-project.org, the R foundation, Austria, Vienna).

## 3. Results

Table 1 shows the baseline data of the patients. No significant differences were observed in corneal power and thickness, ACD, and intraocular pressure (all *p* > 0.11); however, significant differences were observed in SER, AL, and corneal astigmatism (all *p* < 0.001) between the untreated and treated eyes. Excluding the anisometropia value and interocular AL difference (both *p* < 0.001) among the three groups in the treatment eyes, other parameters including SER, AL, corneal power, thickness and astigmatism, ACD, and intraocular pressure did not show a significant difference (all *p* > 0.29). Excluding SER, AL, corneal astigmatism, anisometropia value, and interocular AL difference (all *p* < 0.001 except *p* = 0.01 for astigmatism) among the three groups in the untreated eyes, the other parameters did not show a significant difference (all *p* > 0.21).

**Table 1:** Baseline characteristics of the study participants grouped by the SER of untreated eyes [mean ± SD or median (Q1, Q3)].

	**Treated eyes**	**Untreated eyes**	***p*-value**	**Treated eyes**				**Untreated eyes**			
	**All**	**All**		**Low myopia in the untreated eyes**	**Emmetropia in the untreated eyes**	**Low hyperopia in the untreated eyes**	***p*** **-value**	**Low myopia**	**Emmetropia**	**Low hyperopia**	***p*** **-value**
Number of eyes (*N*)	148	148		37	76	35		37	76	35	
Age (year)	11.5 ± 1.9			11.1 ± 2.2	11.6 ± 1.9	11.6 ± 1.8	0.40				
Sex (male, *n* and %)	65 (43.9%)			18 (48.6%)	35 (46.1%)	12 (34.3%)	0.41				
SER(D)	−2.50 (−1.75, −3.00)	0.00 (+0.38, −0.41)	< 0.001	−2.70 ± 0.94	−2.41 ± 0.95	−2.46 ± 0.78	0.29	−0.75 (−0.56, −0.88)	0.00 (0.00, −0.25)	+0.75 (+1.00,+0.62)	< 0.001
AL (mm)	24.67 ± 0.73	23.58 ± 0.67	< 0.001	24.76 ± 0.76	24.65 ± 0.76	24.62 ± 0.66	0.70	23.90 ± 0.62	23.57 ± 0.67	23.28 ± 0.59	< 0 .001
Anisometropia value (D)	2.45 ± 1.04			1.90 ± 0.89	2.36 ± 0.95	3.34 ± 0.87	< 0.001				
Interocular AL difference (mm)	1.09 ± 0.45			0.86 ± 0.39	1.08 ± 0.45	1.35 ± 0.38	< 0.001				
Corneal power (D)	42.55 ± 1.38	42.45 ± 1.33	0.50	42.36 ± 1.56	42.69 ± 1.41	42.45 ± 1.07	0.53	42.20 ± 1.40	42.62 ± 1.40	42.35 ± 1.04	0.21
Corneal astigmatism (D)	1.17 ± 0.42	1.45 ± 0.61	< 0.001	1.27 ± 0.47	1.15 ± 0.41	1.13 ± 0.40	0.36	1.71 ± 0.68	1.39 ± 0.48	1.25 ± 0.67	0.01
Central corneal thickness (μm)	534.1 ± 37.0	531.8 ± 36.3	0.63	534.4 ± 42.8	531.1 ± 31.2	537.8 ± 39.6	0.92	528.7 ± 38.5	531.9 ± 30.9	535.1 ± 42.2	0.74
Intraocular pressure (mmHg)	15.72 ± 2.61	16.04 ± 2.90	0.45	15.50 ± 2.52	15.91 ± 2.80	15.58 ± 2.35	0.82	15.88 ± 2.75	16.30 ± 3.06	15.72 ± 2.78	0.75
Anterior chamber depth (mm)	3.72 ± 0.27	3.67 ± 0.26	0.11	3.72 ± 0.21	3.75 ± 0.23	3.67 ± 0.36	0.80	3.67 ± 0.25	3.66 ± 0.24	3.60 ± 0.25	0.75

SER, spherical equivalent refraction; AL, axial length.

Among the untreated eyes, the uncorrected VA of the low hyperopia and emmetropia subgroups was 0 (LogMAR charts) or better, and 12 eyes (32.4%) in the low myopia subgroup exhibited an uncorrected VA > 0.1, with an average VA of 0.25 ± 0.17. Among the 37 eyes in the low myopia subgroup, 13 eyes had a sphere diopter between −0.50 and −1.00 D and a cylinder diopter between 0 and −0.75 D, 10 eyes had a sphere diopter of −0.25 D and a cylinder diopter between −0.50 and −1.25 D, 13 eyes had a sphere diopter of 0 D and a cylinder diopter between −1.00 and −2.00 D, and one eye had a sphere diopter of +0.50 D and a cylinder diopter of −2.00 D. The average SER [median (Q1, Q3)] was −2.50 D (−1.75 D, −3.00 D) and 0.00 D (+0.38 D, −0.41 D) in the treated and untreated eyes, respectively (*p* < 0.001; Table 1). In the untreated eyes, the average SER values were −0.75 D (−0.56 D, −0.88 D), 0.00 D (0.00 D, −0.25 D), and +0.75 D (+1.00 D, +0.62 D) in low myopia (*n* = 37), emmetropia (*n* = 76), and low hyperopia (*n* = 35) subgroups; respectively.

After 1-year follow-up, axial elongation was 0.14 ± 0.18 mm and 0.39 ± 0.27 mm in all treated and untreated eyes, respectively (independent sample *t*-test, *p* < 0.001). The interocular difference in AL decreased from 1.09 ± 0.45 mm at baseline to 0.84 ± 0.52 mm at 1 year (independent sample *t*-test, *p* < 0.001) and the reduction in the interocular AL difference (equal to binocular AL difference at baseline minus binocular AL difference at 1 year after treatment) was 0.25 ± 0.30 mm (Table 2). After univariate analysis, the reduction in the interocular AL difference was correlated with age (β = −0.02, *p* = 0.04), anisometropia value (β = 0.05, *p* = 0.03), and refractive state of the untreated eyes (all *p* < 0.05), but independent of sex (*p* = 0.76). In the untreated eyes, AL changes in low myopia, emmetropia, and low hyperopia subgroups were 0.44 ± 0.25 mm, 0.35 ± 0.24 mm, and 0.41 ± 0.33 mm, respectively (ANOVA, *p* = 0.11). AL changes in the corresponding eyes from the treated eyes were 0.14 ± 0.18 mm, 0.15 ± 0.17 mm, and 0.13 ± 0.21 mm (ANOVA, *p* = 0.92).

**Table 2:** Change in AL of the two eyes in the three subgroups [mean ± SD or median (Q1, Q3)].

	**Treated eyes**	**Untreated eyes**	***p*-value**	**Treated eyes**				**Untreated eyes**			
	**All**	**All**		**Low myopia in the untreated eyes**	**Emmetropia in the untreated eyes**	**Low hyperopia in the untreated eyes**	***p*** **-value**	**Low myopia**	**Emmetropia**	**Low hyperopia**	***p*** **-value**
Number of eyes (*N*)	148	148		37	76	35		37	76	35	
SER at baseline (D)	−2.50 (−1.75, −3.00)	0.00 (+0.38,−0.41 )	< 0.001	−2.70 ± 0.94	−2.41 ± 0.95	−2.46 ± 0.78	0.29	−0.75 (−0.56, −0.88)	0.00 (0.00, −0.25)	+0.75 (+1.00,+0.62)	< 0.001
AL at baseline (mm)	24.67 ± 0.73	23.58 ± 0.67	< 0.001	24.76 ± 0.76	24.65 ± 0.76	24.62 ± 0.66	0.70	23.90 ± 0.62	23.57± 0.67	23.28 ± 0.59	< 0.001
AL at 1 year (mm)	24.81 ± 0.72	23.97 ± 0.73	< 0.001	24.89 ± 0.78	24.80 ± 0.75	24.75 ± 0.63	0.69	24.34 ± 0.72	23.92± 0.71	23.69 ± 0.65	< 0.001
Change in AL at 1 year (mm)	0.14 ± 0.18	0.39 ± 0.27	< 0.001	0.14 ± 0.18	0.15 ± 0.17	0.13 ± 0.21	0.92	0.44 ± 0.25	0.35 ± 0.24	0.41 ± 0.33	0.11
AL difference (treated minus untreated eyes at baseline)#	1.09 ± 0.45			0.86 ± 0.39	1.08 ± 0.45	1.35 ± 0.38	< 0.001				
AL difference (treated minus untreated eyes at 1 year)※	0.84 ± 0.52			0.55 ± 0.47	0.88 ± 0.50	1.06 ± 0.51	< 0.001				
Reduction of AL difference (# minus※)	0.25 ± 0.30			0.30 ± 0.25	0.20 ± 0.28	0.28 ± 0.37	0.17				

SER, spherical equivalent refraction; AL, axial length.^#, ※^The symbol for the horizontal column title. This makes the bottom column of the table more concise when calculating the difference between the two variables.

Multivariate linear regression analyses showed no significant differences in axial elongation in the treated eyes when the corresponding eyes from the untreated eyes had low myopia, emmetropia, or low hyperopia (all *p* > 0.05; adjusting for age, sex, and anisometropia values) and significant differences in axial elongation only between low myopia and emmetropia subgroups of the untreated eyes (β = 0.09, *p* = 0.04; *p* > 0.05 between other subgroups; adjusting for age, sex, corneal astigmatism, and anisometropia). The axial elongation in the low myopia subgroup was 0.09 mm greater than that in the emmetropia subgroup of the untreated eyes (Table 3). Moreover, the axial elongation in the untreated eyes was greater than that in the treated eyes (β = 0.23, *p* < 0.001; adjusting for baseline AL, corneal power, and anterior chamber depth). The reduction in the interocular AL difference was significantly associated with age (β = −0.04, *p* = 0.009), anisometropia value (β = 0.09, *p* = 0.003), and refractive state of the untreated eyes (β_low myopia vs. emmetropia subgroups_ = −0.12, *p* = 0.045; *p* > 0.05 between other subgroups) after multivariate linear regression analyses.

**Table 3:** Multivariate linear regression analyses of change in axial length in the three subgroups based on the SER of untreated eyes.

	**Subgroups**	**Non-adjusted model**		**Sex and age-adjusted model**		**Sex, age, corneal astigmatism, and anisometropia value-adjusted model**	
		**β, 95% CI**	***p*-value**	**β, 95% CI**	***p*-value**	**β, 95% CI**	***p*-value**
Treated eyes	Emmetropia in the untreated eyes	0		0			
	Low myopia in the untreated eyes	−0.02 (−0.10, 0.05)	0.54	−0.03 (−0.10, 0.04)	0.44	−0.01 (−0.08, 0.07)	0.86
	Low hyperopia in the untreated eyes	−0.01 (−0.09, 0.06)	0.72	−0.03 (−0.10, 0.03)	0.33	−0.04 (−0.11, 0.03)	0.25
Untreated eyes	Emmetropia	0		0		0	
	Low myopia	0.09 (−0.02, 0.19)	0.10	0.08 (0.01, 0.15)	0.04	0.09 (0.01, 0.17)	0.04
	Low hyperopia	0.06 (−0.04, 0.17)	0.25	0.04 (−0.06, 0.14)	0.41	0.06 (−0.04, 0.16)	0.22

SER, spherical equivalent refraction.

Of all children, 15 children exhibited slight peripheral corneal epithelial staining, but no obvious symptoms of irritation in the early days of wearing the OK lens. However, the eyes showed complete recovery several days after discontinuing the OK lens, with or without 0.1% sodium hyaluronate eye drops. The eyes wearing the OK lens showed no other serious complications, such as corneal stromal infiltration or corneal ulceration.

## 4. Discussion

This retrospective cohort study included myopic anisometropic children who wore the OK lens only in the more myopic eye. It was observed that axial elongation in the eyes wearing the OK lenses (treated eyes) was significantly slower than that in the uncorrected contralateral eyes (untreated eyes), and the interocular AL differences decreased after 1-year follow-up. The refractive state (low myopia, emmetropia, and low hyperopia) of the untreated contralateral eyes did not affect the axial elongation of the myopic eyes wearing the OK lens. The AL of the untreated contralateral eyes increased faster in the low myopia subgroup than in the emmetropia subgroup.

The results that unilateral OK lenses effectively suppressed axial elongation in the more myopic eyes and reduced interocular AL differences in children with myopic anisometropia were consistent with those of five other studies (Table 4) (11–15). The reduction in interocular AL differences in anisometropic children following treatment with monocular OK lenses may be due to the influence of peripheral defocus induced by OK wear (23) rather than the natural development laws of anisometropia. In a 1-year retrospective study, Long et al. (13) divided patients with UMA into the treated eyes (wearing OK lens only in the myopic eyes) group and the spectacle group, which was matched for age, refraction, and AL. They observed that changes in AL in the myopic eyes (treated eyes) were lower than that in the non-myopic eyes (treated eyes) and both the eyes from the spectacle group. However, there was no statistically significant difference in change in AL between the non-myopic eyes of the two groups and between the two eyes in the spectacle group. The most probable mechanism of action of the OK lens was increased myopic defocus in the peripheral retina, thereby decreasing the tendency of axial elongation (23). Although these studies and the present study reported that AL differences between the uncorrected contralateral eyes and the eyes wearing the OK lenses decreased in children with UMA, reduction in the binocular AL differences from baseline to 1 year after treatment were slightly different among various studies [0.15 mm (11), 0.31 mm (12), 0.29 mm (13), 0.19 mm (14), 0.36 mm (15), and 0.25 mm in the present study]. These differences in the values may be attributed to the differences in study populations, baseline characteristics, anisometropia values, genetic factors such as the number of myopic parents (24), and other possible factors. This study observed that younger children with low anisometropia values in the low myopia subgroup exhibited a greater reduction in the binocular AL differences.

**Table 4:** Change in AL of both eyes in anisometropia children who wore OK lenses in the more myopic eyes in different studies.

**References**	**Number**	**Follow-up time (year)**	**Age (year)**	**OK lens group (treated eyes)**			**Control group (untreated eyes)**			**Baseline SER difference**	**Reduction of AL difference (mm/year)**
				**Baseline SER (D)**	**Baseline AL (mm)**	**AL elongation rate (mm/year)**	**Baseline SER (D)**	**Baseline AL (mm)**	**AL elongation rate (mm/year)**		
Chen et al. (12)	25	1.93	11.2 ± 1.9 (9–15)	−2.32 ± 0.94 (−1.00 to −4.00)	24.56 ± 0.72	0.08 ± 0.15	+0.24 ± 0.50 (+1.75 to −0.50)	23.47 ± 0.65	0.39 ± 0.32	2.56	0.31
Long et al. (13)	79	1.0	11.46 ± 1.76 (8–14)	−2.60 ± 1.14	24.79 ± 0.90	0.05 ± 0.19	+0.10 ± 0.42 (+1.50 to −0.50)	23.64 ± 0.78	0.34 ± 0.21	2.70	0.29
Fu et al. (14)	27	1.0	12.41 ± 2.32 (8–18)	−2.83 ± 1.37 (−1.25 to −6.50)	24.68 ± 0.70	0.11 ± 0.19	−0.07 ± 0.35 (+0.50 to −0.50)	23.51 ± 0.72	0.30 ± 0.28	2.76	0.19
Tsai et al. (11)	31	2.01	12.32 ± 3.07 (>8)	−2.73 ± 0.95	24.15 ± 1.13	–	−0.37 ± 0.50 (+0.50 to −0.50)	23.32 ± 1.04	–	2.36	0.15 (1st year) 0.09 (2nd year)
Xu et al. (15)	47	1.28	12.35 ± 2.37 (8–16)	−2.31 ± 1.16 (−1.00 to −6.00)	24.54 ± 0.94	About 0.11	0.15 ± 0.49 (+1.50 to −0.50)	23.46 ± 0.74	About 0.35	2.46	0.36 (1st year) 0.14 (2nd year)
**Current study**											
All	148	1.18	11.5 ± 1.9 (8–14)	−2.50 ± 0.91 (−1.00 to −4.38)	24.67 ± 0.73	0.14 ± 0.18	−0.05 ± 0.65 (+1.88 to −1.13)	23.58 ± 0.67	0.39 ± 0.27	2.45	0.25
Low myopia	37	1.11	11.1 ± 2.2 (8–14)	−2.70 ± 0.94 (−1.00 to −4.38)	24.76 ± 0.76	0.14 ± 0.18	−0.80 ± 0.20 (−0.50 to −1.13)	23.90 ± 0.62	0.44 ± 0.25	1.90	0.30
Emmetropia	76	1.22	11.6 ± 1.9 (8–14)	−2.41 ± 0.95 (−1.00 to −4.25)	24.65 ± 0.76	0.15 ± 0.17	−0.05 ± 0.20 (+0.38 to −0.38)	23.57 ± 0.67	0.35 ± 0.24	2.36	0.20
Low hyperopia	35	1.02	11.6 ± 1.8 (8–14)	−2.46 ± 0.78 (−1.00 to −4.10)	24.62 ± 0.66	0.13 ± 0.21	0.88 ± 0.37 (+1.88 to +0.50)	23.28 ± 0.59	0.41 ± 0.33	3.34	0.28

SER, spherical equivalent refraction; AL, axial length.

In the present study, the refractive state (low myopia, emmetropia, and low hyperopia) of the untreated contralateral eyes did not affect the change in AL of the more myopic eyes wearing the OK lens. Similar results have not been reported in any of the previous studies. Clinically, regardless of the refractive state of the untreated contralateral eye (low myopia, emmetropia, or low hyperopia), wearing the OK lens was a good option after the diagnosis of unilateral myopia since it could provide optical correction and also inhibit axial elongation in the myopic eyes, resulting in reduced AL difference between the two eyes.

The current study observed that AL in low-myopic eyes grew faster than emmetropic eyes in untreated eyes. The difference in axial elongation among the untreated contralateral eyes at different refractive states when the myopic eye was wearing the OK lens may relate to several factors. First, it is not related to the OK lens but may relate to the change in peripheral refraction (myopic refraction) induced by midperipheral corneal steeping induced by OK wear (23). Long et al. (13) reported no significant difference in axial elongation between emmetropic and low hyperopic untreated contralateral eyes regardless of whether the myopic eye had OK lenses or spectacles. However, there was a slight difference in the SER range in untreated contralateral eyes between the study by Long et al. (13) (+1.50 D to −0.50 D) and the present study (+1.88 D to −1.13 D). Second, the differences in peripheral refraction (between myopic, emmetropic, or hyperopic eyes of the untreated contralateral eyes) could be related to AL change (25–27). Third, there may be a sympathetic effect between the eyes of the same patient, as seen with other eye conditions. Finally, among the 37 eyes in the low myopia subgroup of the untreated eyes (SER: −0.50 D to −1.13 D), 13 eyes exhibited a sphere diopter of more than −0.50 D, and 19 cases exhibited a cylinder diopter of more than −1.00 D. The uncorrected VA of the untreated eyes was higher than 0 (LogMAR charts). However, these children and their parents or guardians were reluctant to receive any form of intervention and gave consent only for the OK lens in the more myopic eye. Thus, the untreated eyes were in a state of uncorrected myopia or minus astigmatism during the follow-up period. Uncorrected myopia (28, 29) and astigmatism (30, 31) have been shown to hasten the progression of myopia. Therefore, for children with UMA, methods of correcting the eyes with lower myopia such as wearing the OK lens should be adopted to slow down axial elongation and balance the binocular VA when the OK lens is used in the more myopic eye. Chen et al. (12) observed that in children with UMA, the OK lens could also slow down axial elongation in the initially non-myopic eye that developed myopia subsequently. Therefore, further prospective studies should include peripheral refraction assessment (at baseline, with and without OK lens wear, and after treatment) between other clinical variables.

The present study has some limitations. The sample sizes of the three subgroups were unequal, with more cases in the emmetropia subgroup than in the low hyperopia and low myopia subgroups. However, all patients who met the inclusion criteria at the hospital were included in the study. Moreover, the current study evaluated the effect of the uncorrected eye in monocular OK lens wearing. The uncorrected eyes usually have a low degree of refractive error which may result in small differences of refractive error in the three subgroups and play little impact on axial elongation or change in interocular AL differences. Other limitations were the lack of a spectacle-wearing control group, not collecting genetic factors (number of myopic parents) from the children of participants, not measuring and analyzing the differences in peripheral refraction, the retrospective nature of the study, and the short follow-up time. Further prospective investigations with long-term follow-up and studies including control group design and peripheral refraction analysis should be conducted to confirm the findings of the present study.

In conclusion, among children with myopic anisometropia aged 8–14 years, wearing OK lenses in the more myopic eyes without any intervention in the contralateral eyes (SER: +2.00 to −1.25 D) could effectively slow down axial elongation in more myopic eyes, thereby reducing the interocular AL difference. The refractive state (low myopia, emmetropia, or low hyperopia) of the untreated eyes did not affect the axial elongation of more myopic eyes wearing the OK lens. In the untreated eyes, AL increased faster in the low myopia subgroup than in the emmetropia subgroup. Clinically, one of the reasonable treatment methods for children with UMA is wearing the OK lenses in the more myopic eyes and a regular follow-up of the untreated eyes if they have emmetropia or very low hyperopia. Appropriate treatment measures employed when the untreated eyes become myopic can balance the VA in both eyes, control axial elongation, and reduce the interocular AL difference. These could reduce the incidence of strabismus and amblyopia and could better establish binocular visual function.

## Data availability statement

The raw data supporting the conclusions of this article will be made available by the authors, without undue reservation.

## Ethics statement

The studies involving humans were approved by Medical Ethics Committee of the First Affiliated Hospital of Zhengzhou University. The studies were conducted in accordance with the local legislation and institutional requirements. Written informed consent for participation in this study was provided by the participants' legal guardians/next of kin.

## Author contributions

JQ: Conceptualization, Data curation, Writing—review & editing, Investigation, Project administration, Writing—original draft. HQ: Investigation, Writing—review & editing, Data curation, Supervision. NJ: Writing—review & editing. TL: Writing—review & editing, Data curation. HM: Data curation, Writing—review & editing. MS: Data curation, Writing—review & editing. SY: Data curation, Writing—review & editing. CM: Data curation, Writing—review & editing, Formal analysis. AF: Data curation, Formal analysis, Writing—review & editing, Conceptualization, Methodology.
